# Integrating somatic mutation profiles with structural deep clustering network for metabolic stratification in pancreatic cancer: a comprehensive analysis of prognostic and genomic landscapes

**DOI:** 10.1093/bib/bbad430

**Published:** 2023-12-01

**Authors:** Min Zou, Honghao Li, Dongqing Su, Yuqiang Xiong, Haodong Wei, Shiyuan Wang, Hongmei Sun, Tao Wang, Qilemuge Xi, Yongchun Zuo, Lei Yang

**Affiliations:** College of Bioinformatics Science and Technology, Harbin Medical University, Harbin 150081, China; College of Bioinformatics Science and Technology, Harbin Medical University, Harbin 150081, China; College of Bioinformatics Science and Technology, Harbin Medical University, Harbin 150081, China; College of Bioinformatics Science and Technology, Harbin Medical University, Harbin 150081, China; College of Bioinformatics Science and Technology, Harbin Medical University, Harbin 150081, China; College of Bioinformatics Science and Technology, Harbin Medical University, Harbin 150081, China; College of Bioinformatics Science and Technology, Harbin Medical University, Harbin 150081, China; College of Bioinformatics Science and Technology, Harbin Medical University, Harbin 150081, China; The State Key Laboratory of Reproductive Regulation and Breeding of Grassland Livestock, College of Life Sciences, Inner Mongolia University, Hohhot 010070, China; The State Key Laboratory of Reproductive Regulation and Breeding of Grassland Livestock, College of Life Sciences, Inner Mongolia University, Hohhot 010070, China; Digital College, Inner Mongolia Intelligent Union Big Data Academy, Inner Mongolia Wesure Date Technology Co., Ltd. Hohhot 010010, China; Inner Mongolia International Mongolian Hospital, Hohhot 010065, China; College of Bioinformatics Science and Technology, Harbin Medical University, Harbin 150081, China

**Keywords:** pancreatic cancer, metabolic pathway, somatic mutation, structural deep clustering network

## Abstract

Pancreatic cancer is a globally recognized highly aggressive malignancy, posing a significant threat to human health and characterized by pronounced heterogeneity. In recent years, researchers have uncovered that the development and progression of cancer are often attributed to the accumulation of somatic mutations within cells. However, cancer somatic mutation data exhibit characteristics such as high dimensionality and sparsity, which pose new challenges in utilizing these data effectively. In this study, we propagated the discrete somatic mutation data of pancreatic cancer through a network propagation model based on protein–protein interaction networks. This resulted in smoothed somatic mutation profile data that incorporate protein network information. Based on this smoothed mutation profile data, we obtained the activity levels of different metabolic pathways in pancreatic cancer patients. Subsequently, using the activity levels of various metabolic pathways in cancer patients, we employed a deep clustering algorithm to establish biologically and clinically relevant metabolic subtypes of pancreatic cancer. Our study holds scientific significance in classifying pancreatic cancer based on somatic mutation data and may provide a crucial theoretical basis for the diagnosis and immunotherapy of pancreatic cancer patients.

## INTRODUCTION

Pancreatic cancer is a highly aggressive malignancy originating from pancreatic tissues. It is characterized by rapid cell proliferation, invasion into surrounding tissues and early metastasis, resulting in a poor prognosis for patients [1–4]. In 2023, an estimated 64 050 new cases of pancreatic cancer are expected to be diagnosed in the United States, making it one of the most prevalent cancer types in the country [5]. Unfortunately, pancreatic cancer remains a leading cause of cancer-related deaths, with an estimated 50 550 individuals projected to die from the disease in the same year [5, 6]. The diagnosis of pancreatic cancer poses challenges due to the lack of specific early symptoms, often resulting in advanced-stage detection. The 5-year survival rate for pancreatic cancer remains low, with only approximately 10% of patients surviving beyond 5 years after diagnosis. Therefore, there is a pressing need for researchers to develop approaches aimed at improving early detection methods and developing more effective treatment strategies for pancreatic cancer, ultimately enhancing the survival rates of patients with this disease [7]. Pancreatic cancer exhibits heterogeneity across multiple dimensions, including genetic, molecular and clinical characteristics. Classifying pancreatic cancer patients into distinct molecular subtypes holds the potential to enhance outcome predictions, guide therapy selection and deepen our understanding of heterogeneity.

Cellular metabolism refers to the set of biochemical processes that occur within cells to acquire, convert and utilize energy and nutrients for various cellular activities [8, 9]. Metabolic reprogramming is widely recognized as a hallmark of cancer and holds immense potential for cancer diagnosis, prognosis and therapeutic interventions [10, 11]. However, research exploring the characterization of cancer metabolism is still relatively limited.

In recent years, with the advancement of cancer genomics research, researchers have discovered that cancer development is often driven by the accumulation of somatic mutations within cells [12–15]. Somatic mutations refer to mutations occurring in non-germline somatic cells, which do not alter hereditary information in subsequent generations but can lead to genetic changes within contemporary cells. Normal somatic cells in humans typically contain a significant number of somatic mutations. As individuals age, somatic mutations continue to accumulate within cells. When the accumulation reaches a certain threshold, it can induce changes in tissue function and morphology, ultimately leading to the development of cancer. Therefore, somatic mutations serve as crucial driving factors in cancer initiation and progression. Nevertheless, cancer somatic mutation data exhibit characteristics such as high dimensionality and sparsity, which pose new challenges in utilizing this data effectively. Therefore, it is worthwhile to investigate methods to tackle the sparsity issue in somatic mutation data and enable the application of traditional mRNA expression profiling methods to analyze mutation profiling [16–24].

Recently, there has been a significant emergence of deep clustering methods, fueled by the advancements in deep neural networks [25]. These methods have demonstrated remarkable performance, positioning them as the prevailing clustering approaches within the field, setting the standard for state-of-the-art performance [26]. Despite the achievements in the deep clustering methods, it frequently fails to consider the structure of data when acquiring the representation, instead focusing the feature representation of the data itself [27]. The structure of the data reveals latent similarities among samples, offering valuable guidance for learning the representation. Hence, the integration of structural information into the deep clustering process, known as structural deep clustering, has garnered growing attention [27–29]. Among various structural deep clustering models, the structural deep clustering network (SDCN) has attracted considerable research attention due to its remarkable capability of incorporating structural information into the deep clustering process [28]. Compared to other state-of-the-art clustering algorithms, SDCN has exhibited superior clustering performance across various real-world datasets. Therefore, the utilization of SDCN is essential in incorporating the structural relationships among pancreatic cancer patients into deep clustering algorithms to effectively classify them into clinically significant subtypes.

In 2015, Cheng *et al.* developed the Memorial Sloan Kettering-Integrated Mutation Profiling of Actionable Cancer Targets (MSK-IMPACT), a hybridization capture-based next-generation sequencing assay, to identify somatic mutations, copy number alterations and structural variants in tumor samples [30]. In the validation datasets, Cheng *et al.* demonstrated that the MSK-IMPACT platform can effectively and accurately detect known somatic mutations, copy number alterations and structural variants confirmed by other methods. In 2017, Cheng *et al.* conducted a further evaluation of the MSK-IMPACT platform to detect genetic alterations in 76 genes associated with cancer predisposition syndromes [31]. In a dataset of 233 cancer patient samples confirmed by alternative methods, they observed that the MSK-IMPACT platform can identify germline variants contributing to cancer predisposition and simultaneously detect both somatic and germline alterations. In 2021, Fiala *et al.* presented the results of 751 patients with pediatric solid tumors via the MSK-IMPACT platform [32]. Their study encompasses an exploration of the frequency and range of pathogenic or likely pathogenic (P/LP) germline variants in genes associated with cancer predisposition and the relationships between germline status and somatic genetic characteristics, as well as the subsequent clinical application of this information. In 2022, Nguyen *et al.* gathered an extensive clinico-genomic cohort from MSKCC, comprising over 25 000 metastatic cancer patients spanning 50 distinct tumor types [33]. Within this extensive metastatic cancer cohort, Nguyen *et al.* observed associations between somatic alterations and metastasis, as well as correlations between chromosomal instability and metastatic burden. The somatic mutation profiles of various cancer types generated by the MSK-IMPACT platform offer the opportunity to classify these cancers into distinct subtypes.

**Figure 1:**
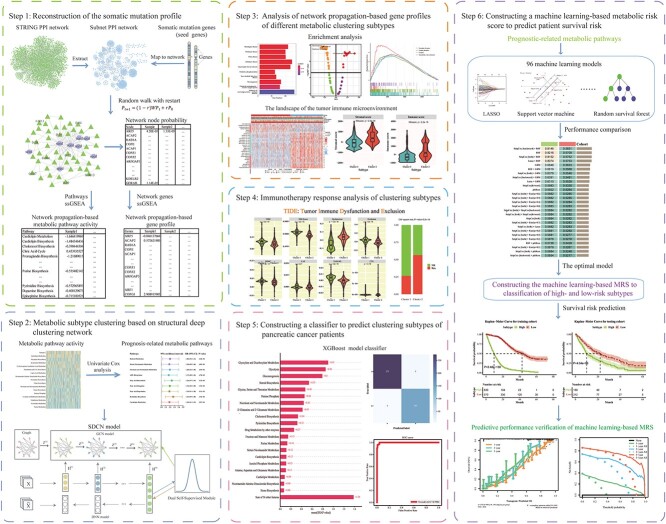
The workflow of our research.

In this study, we present a novel approach that integrates gene mutation profiles with structural deep clustering for the classification of pancreatic cancer subtypes. We performed an analysis on the somatic mutational profiles of 1942 pancreatic cancer patients enrolled at the Memorial Sloan Kettering Cancer Center (MSKCC) [30–33]. The somatic mutation data of 1942 unique pancreatic cancer patients were obtained from the work of Nguyen *et al.* [33]. In the work of Nguyen *et al.*, they collected a large clinico-genomic cohort from the MSKCC that covering more than 25 000 cancer patients with metastasis across 50 tumor types. These cancer patients were sequenced at the MSKCC between 18 November 2013 and 18 August 2021. To improve data quality, we utilized the network propagation algorithm to transform the binary vectors, which represent somatic mutations, into continuous vectors that reflect the mutational status of all patients with pancreatic cancer. We applied the smoothed somatic mutation profile to perform single-sample gene set enrichment analysis (ssGSEA) [34] and determined the activity levels of 114 metabolic pathways for each patient with pancreatic cancer. Based on the activity levels of metabolic pathways using the SDCN method, we classified patients with pancreatic cancer into two clinically distinguishable subtypes, which exhibited significant differences in prognosis, immunogenomics landscape, therapeutic benefits and other factors. Our study demonstrated the utility of using solely somatic mutation profiles to identify molecular subtypes of pancreatic cancer. Furthermore, this approach represents a crucial step toward classifying other cancer patients who possess only somatic mutation data in future studies. Figure 1 illustrates the research workflow.

## MATERIAL AND METHOD

### Publicly available data collection

The pancreatic cancer cohort containing somatic mutation profiles, genomic data and clinical data were obtained from the work of Nguyen *et al.* [33]. In their research, Nguyen *et al.* curated an extensive clinico-genomic dataset from MSKCC, encompassing over 25 000 metastatic cancer cases across 50 distinct tumor types. These cancer cases were sequenced at MSKCC during the period from 18 November 2013, to 18 August 2021. These data included information on overall survival times, overall survival status, age, sex, curated subtype, sample type, metastatic events at the patient level, tumor purity, tumor mutational burden (TMB), fraction of genome altered (FGA), MSIsensor score and MSIsensor type. A total of 1942 pancreatic cancer samples were downloaded from the cBioPortal for Cancer Genomics (https://www.cbioportal.org/study?id=msk_met_2021), which is a renowned repository for cancer-related data.

It is important to note that these samples corresponded to 1942 unique pancreatic cancer patients who were sequenced at the MSKCC between 18 November 2013 and 18 August 2021 [33]. The sequencing process carried out at MSKCC ensured the comprehensive analysis of the patients’ genetic and clinical information, contributing to a more detailed understanding of pancreatic cancer and its associated factors. Three generations of the MSK-IMPACT panel were employed to identify genetic alterations in the pancreatic tumors. These panels consisted of 341 genes (utilized for 29 patients), 410 genes (utilized for 493 patients) and 468 genes (utilized for 1468 patients). The inclusion of these comprehensive gene panels facilitated the detection of various genetic alterations associated with pancreatic tumors.

In this study, we excluded data with no overall survival time from pancreatic cancer patients and obtained a dataset of 1933 pancreatic cancer patients. For this pancreatic cancer dataset, we divided the patients into training and testing cohorts in a certain proportion. The specific method involved generating random numbers that follow a standard normal distribution for the patients in the pancreatic cancer cohort and sorting them in descending order. We selected the top 1500 cancer patients based on the randomly generated numbers as the training cohort, while the remaining 433 cancer patients formed the testing cohort.

Furthermore, the collection of somatic mutation profiles, genomic data and clinical data extended beyond pancreatic cancer to include other cancer types. Specifically, cohorts for appendiceal cancer (193 patients), bladder cancer (1141 patients), breast cancer (2526 patients), colorectal cancer (3537 patients), endometrial cancer (1308 patients), head and neck cancer (397 patients), hepatobiliary cancer (865 patients), melanoma (1127 patients), non-small cell lung cancer (4605 patients), prostate cancer (1951 patients) and thyroid cancer (400 patients) were assembled. The data for these cohorts were also obtained from the work of Nguyen *et al.* by cBioPortal database (https://www.cbioportal.org/study?id=msk_met_2021), a reputable repository for cancer-related information. Similar to the pancreatic cancer cohort, all patients included in these cohorts were subjected to sequencing at the MSKCC between 18 November 2013 and 18 August 2021. This standardized approach ensured consistency and comparability across the different cancer types, enabling comprehensive analyses and inter-cancer comparisons.

### Preparation of the protein–protein interaction network

The protein–protein interactions in humans were extracted from the STRING database (version 11.5) [35], which can be accessed at https://string-db.org/. To ensure the utilization of dependable human protein–protein interactions, only links with interaction scores exceeding 700 were chosen for subsequent analysis. Consequently, a network comprising 16 134 genes connected by 474 722 interactions was generated for further examination through network propagation analysis. The mutated genes in each pancreatic cancer patient were mapped to nodes on the STRING network to facilitate network propagation.

### The network propagation algorithm

The random walk with restart (RWR) algorithm is a graph-based method used for network propagation and ranking in protein–protein interaction networks and other biological networks [36]. In the RWR algorithm, it simulates a walker starting from the given node(s) and transitioning to randomly selected neighbor node(s) with a probability parameter denoted as *r*. Let *P*_o_ denote the initial probability distribution vector and *P*_t_ represent a vector where the *i*-th element represents the probability of being at node *i* at time step *t*. The probability distribution at step *t* + *1* can be calculated using the iterative form.


(1) \begin{equation*} {P}_{t+1}=\left(1-r\right){W}_{\mathrm{pr}}+r{P}_0 \end{equation*}

where *W* refers to the column-normalized graph adjacency matrix and *r* represents the restart probability, which takes values as 0.75 in this study. The steady-state probability vector *p*∞ is obtained until the change between *P_t_* and *P*_*t* + 1_ is less than10^−6^.

In the STRING network, the seed nodes consisted of somatic mutation genes specific to each pancreatic cancer sample, while all other genes were assigned a value of zero. Through the application of the network propagation algorithm, the influence of the somatic mutation genes was disseminated across the STRING network. Consequently, the smoothed mutation profiles were generated for the training and testing cohorts, respectively.

### Network propagation–based metabolic pathway activity

In order to investigate the metabolic pathway activity in patients with pancreatic cancer, we selected 114 metabolic pathways and 2752 corresponding genes related to metabolism from the research work conducted by the Rosario team [8, 37]. Then, the ssGSEA [34] implemented in the R package GSVA (version 1.48.1) was utilized to quantify the activity levels of 114 metabolic pathways in the smoothed mutation profile. Through this approach, we can obtain the network propagation–based metabolic pathway activity for the pancreatic cancer training and testing cohorts, respectively.

### Network propagation–based gene profile

Due to the unavailability of the mRNA profile for the 1933 distinct pancreatic cancer patients studied in our research from MSKCC, we constructed the network propagation–based gene profile that aimed to capture potential similarities to the mRNA profile. This network propagation–based gene profile was then utilized for conducting bioinformatics analysis. To generate the network propagation–based gene profile for pancreatic cancer patients, we utilized the ssGSEA algorithm. This algorithm enabled us to project the smoothed profile onto the space encompassing all 16 134 gene sets. Notably, in the process of ssGSEA analysis, each gene within the smoothed mutation profile was treated as an individual gene set. After conducting the ssGSEA analysis, the 16 134 gene normalized values for each pancreatic cancer sample in the network propagation–based gene profile ranged from −3 to 3. In this section, the network propagation–based gene profiles of the training cohort and testing cohort were generated by the ssGSEA algorithm, respectively.

### Clustering metabolic pathways by structural deep clustering network

The SDCN introduces a robust and cohesive framework that effectively integrates the representation of an autoencoder with that of a graph convolution network [28]. This integration is achieved through the systematic delivery of semantic information to the graph convolution network module in a layer-by-layer fashion, enhancing the overall performance and functionality of the network.

In this study, in order to achieve robust clustering subtypes for pancreatic cancer patients, the SDCN was applied. Before clustering, a univariate Cox regression analysis was conducted on the 114 metabolic pathways to identify the pathways that exhibited significant correlations with overall survival within the training cohort. Next, the SDCN was employed to perform clustering on these prognosis-related metabolic pathways. The average silhouette width was then computed to evaluate the optimal cluster within the training cohort.

### Differential gene analysis and functional enrichment analysis

The limma package (version 3.48.3) in R [38] was utilized to process the network propagation-based gene profile, which exhibited similarities to mRNA expression, with the aim of identifying differential genes between distinct subtypes of pancreatic cancer. In order to explore the functions of the identified differential genes, Gene Ontology (GO) and Kyoto Encyclopedia of Genes and Genomes (KEGG) analyses were performed using the clusterProfiler R package (version 4.0.5) in R. The clusterProfiler package in R [39] was employed for conducting overrepresentation analysis and preranked Gene Set Enrichment Analysis (GSEA) [40], as well as visualization of the results. In this study, GSEA was utilized to analyze and visualize gene sets associated with 50 hallmarks [41], 114 metabolic pathways [37] and 17 ImmPort immune cells [42].

### Machine learning–based metabolic risk score

To construct a machine learning–based metabolic risk score (MRS), an integration of 10 classical machine learning algorithms was utilized [43–46]. These algorithms included random survival forest (RSF), least absolute shrinkage and selection operator (LASSO), gradient boosting machine (GBM), survival support vector machine (survival-SVM), ridge regression, supervised principal components (SuperPC), partial least squares regression for Cox (plsRcox), CoxBoost, Stepwise Cox and elastic network (Enet). Among these algorithms, RSF, LASSO, CoxBoost and Stepwise Cox were specifically chosen for their demonstrated ability in feature selection. Integration of these four algorithms with others resulted in 96 kinds of machine learning models. The ensemble learning was used in the process of constructing the machine learning–derived MRS.

In this study, RSF was implemented using the R package randomForestSRC (version 3.2.0). LASSO, ridge regression and Enet were implemented using the R package glmnet (version 4.1-6). GBM was implemented using the R package gbm (version 2.1.8.1). Survival-SVM was implemented using the R package survivalsvm (version 0.0.5). SuperPC was implemented using the R package superpc (version 1.12). plsRcox was implemented using the R package plsRcox (version 1.7.7). CoxBoost was implemented using the R package CoxBoost (version 1.5). The stepwise Cox model was based on the R package survival (version 3.2-11) and the R function step.

The machine learning–derived MRS was then generated through the following steps: (a) prognostic-related metabolic pathways were identified by performing univariate Cox regression analysis on the ssGSEA levels of 114 metabolic pathways within the training cohort. (b) The 96 models, including both ensemble machine learning algorithms and individual machine learning models, were constructed using the prognostic-related metabolic pathways and evaluated through 10-fold cross-validation within the training cohort. (c) The performance of these 96 models was further assessed in the testing cohort. The model demonstrating the highest average C-index across both the training and testing cohorts was identified as the optimal model. Finally, the predicted probability value from the optimal model with either the individual machine learning algorithm or the second machine learning algorithm within the ensemble machine learning algorithm were determined as the machine learning–based MRS.

### Bioinformatics tools and statistical analysis

The Tumor Immune Dysfunction and Exclusion (TIDE) algorithm [47] is a computational tool designed to predict the response to immune checkpoint blockade (ICB) therapy in cancer patients. It aims to identify tumors that are likely to exhibit resistance or poor response to ICB treatment. In this study, we employed the TIDEpy Python package to calculate a range of scores, including the responder, TIDE score, dysfunction score and exclusion score, utilizing the network propagation–based gene profile. By leveraging the network propagation–based gene profile, the calcPhenotype function from the oncoPredict R package (version 0.2) [48] was utilized to assess the chemotherapeutic response in pancreatic cancer patients.

Categorical variables were compared using the chi-squared test, while the Wilcoxon rank-sum test was employed for comparing continuous variables. Prognostic differences between different subtypes were estimated by the Kaplan–Meier analysis, and the statistical significance was estimated by the log-rank test. The univariate Cox regression analysis was employed to evaluate the associations between metabolic pathways and prognosis. In order to examine whether the cluster subtype was independent with other clinical features, multivariable Cox regression analysis was conducted. All statistical analyses in this study were conducted using R software (version 4.2.1) or Python (version 3.9). All statistical tests were two-sided, and a *P*-value < 0.05 was considered statistically significant.

## RESULTS

### Two cluster subtypes were identified from the network propagation–based metabolic pathways in pancreatic cancer training cohort

**Figure 2:**
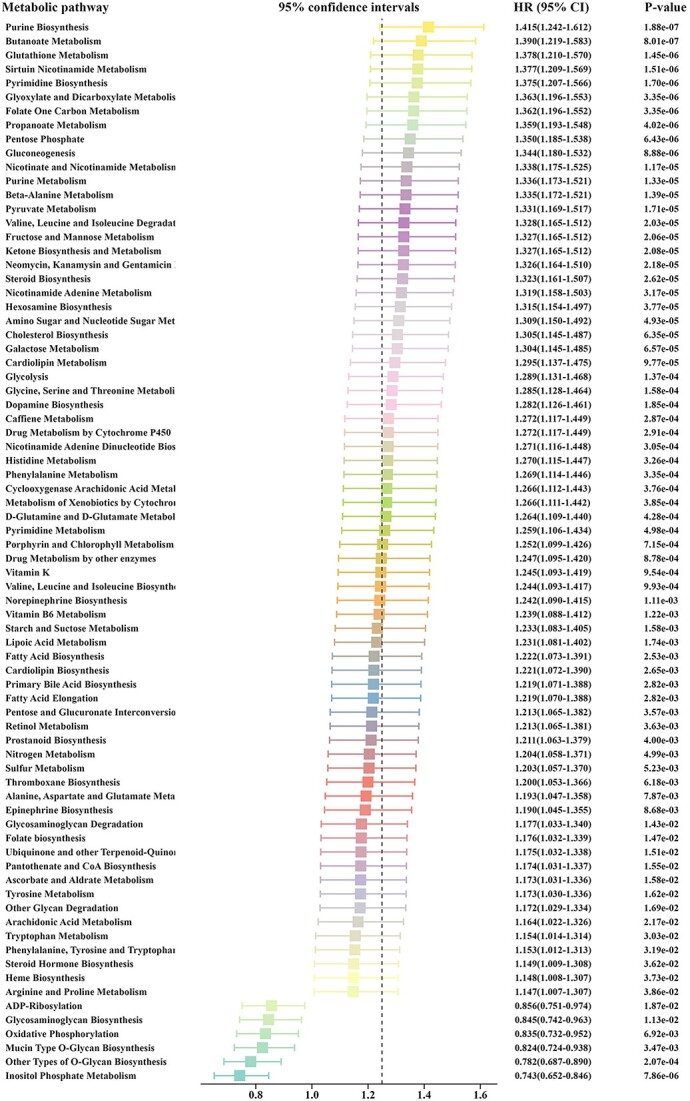
The forest plot illustrates the 95% confidence intervals (CIs), hazard ratios (HRs) and corresponding *P*-values for 76 metabolic pathways within the MSKCC pancreatic cancer training cohort. These statistical values were obtained through univariate Cox regression analysis, providing valuable insights into the associations between the metabolic pathways and pancreatic cancer prognosis.

The activity levels of 114 metabolic pathways were subjected to univariate Cox regression analysis in the training cohort of pancreatic cancer patients. A set of 76 metabolic pathways that exhibited significant associations with overall survival in patients was identified (*P*-value < 0.05; log-rank test). Among these, higher activity levels in six metabolic pathways were associated with longer overall survival, while higher activity levels in the remaining 70 metabolic pathways were associated with shorter overall survival (Figure 2). In recent years, the bile acid metabolism was found to be associated with bad prognosis in breast cancer [49]. The fatty acid metabolism was associated with bad prognosis in colon cancer and breast cancer [50–52]. The metabolic reprogramming and metabolism-related genes can be used as a predictor for prognosis in pancreatic cancer [53, 54]. In 2020, Fernández and colleagues summarized the association between lipid metabolism alterations and prognosis in different types of cancers. They observed that fatty acid–related pathways, such as fatty acid synthesis, fatty acids–related transportation, cholesterol-related pathways, such as cholesterol synthesis, cholesterol-related transportation and lipid transcription, were commonly linked to prognosis in multiple cancer types, including pancreatic cancer [55]. In our study, we discovered that primary bile acid biosynthesis, fatty acid degradation, fatty acid elongation, fatty acid biosynthesis and cholesterol biosynthesis were all significantly associated with a poor prognosis in our MSKCC pancreatic cancer cohort, which was similar to these previous well-conducted studies (Figure 2). In addition, in our previous work [56], using the same 114 metabolic pathways, 62 metabolic pathways, including glycolysis, gluconeogenesis and purine metabolism, were identified to be significantly associated with overall survival in breast cancer. Of the 62 metabolic pathways identified as prognostic in breast cancer, 47 of them were also associated with prognosis in the MSKCC pancreatic cancer cohort (Table S1). Our study demonstrates that analyzing metabolic pathway activity based solely on somatic mutation data from pancreatic cancer can identify metabolic pathways significantly associated with prognosis, similar to those derived from cancer expression profiles. Furthermore, we also observed that, consistent with other studies, most of the metabolic pathways identified in this study are associated with a poor prognosis in pancreatic cancer. To the best of our knowledge, this study represents the first systematic evaluation of metabolic pathway activity using somatic mutation data in pancreatic cancer. Therefore, such conclusions were not reached in previous research.

**Figure 3:**
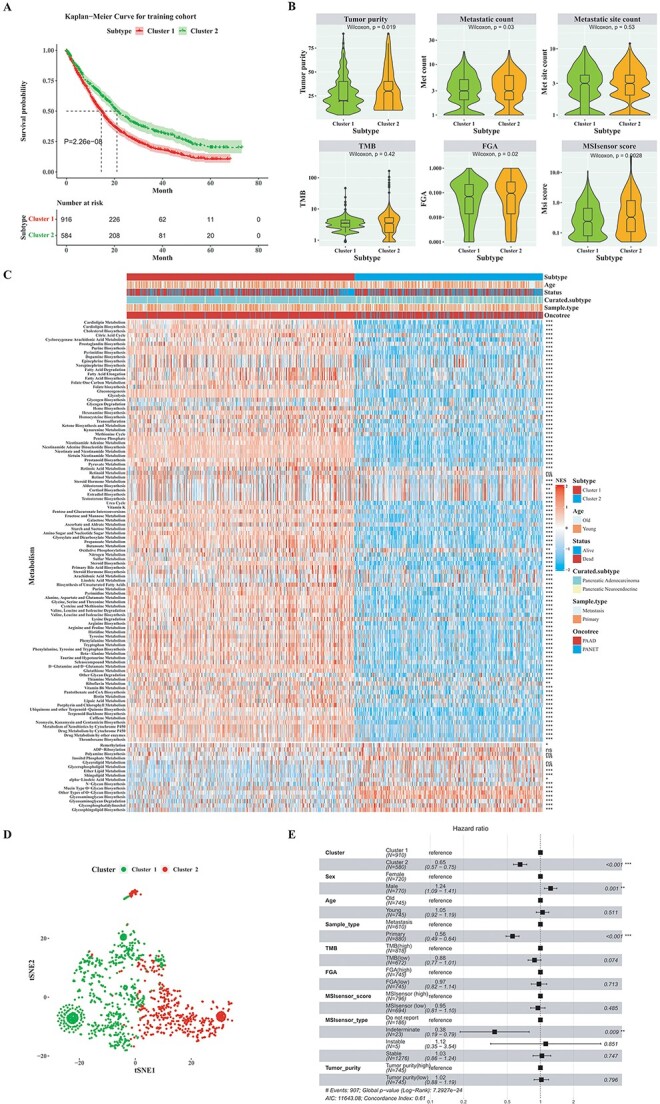
The clinical signature and functional characteristics of the two cluster subtypes in the pancreatic cancer training cohort. (**A**) Kaplan–Meier curves of overall survival with log-rank test for two cluster subtypes. (**B**) The violin plots distribution of tumor purity, metastatic count, metastatic site count, TMB, FGA and MSIsensor score between two cluster subtypes. (Wilcoxon rank-sum test). (**C**) The distribution of activity levels across 114 metabolic pathways, discerning variations between the two cluster subtypes. Statistical significance levels are indicated as follows: ns signifies *P*-value > 0.05, * signifies *P*-value < 0.05, ** signifies *P*-value < 0.01 and *** signifies *P*-value < 0.001, with all values determined through the Wilcoxon rank-sum test. (**D**) The t-SNE analysis projected all pancreatic cancer samples onto two-dimensional spatial coordinates, demonstrating good discrimination for two cluster subtypes. (**E**) A forest plot of multivariate Cox regression analysis of including the cluster subtypes and clinical annotations for pancreatic cancer patients.

According to the activity levels of 76 prognostic-related metabolic pathways assessed by ssGSEA, the subtypes of pancreatic cancer were identified in the training cohort using the SDCN algorithm. The average silhouette coefficient of the SDCN algorithm indicated that the optimal number was obtained when *k* = 2 in the training cohort (Figure S1A). The silhouette width was also calculated for each sample within every cluster subtype (Figure S1B). To determine the association between cluster subtypes and prognosis in pancreatic cancer patients, Kaplan–Meier survival analysis was performed. The log-rank test yielded a *P*-value of 2.26E-8, indicating that pancreatic cancer patients with different cluster subtypes had distinct overall survival probabilities (Figure 3A). As depicted in Figure 3A, patients in cluster 2 subtype exhibited significantly longer overall survival compared to those in cluster 1 subtype. We further compared several clinicopathological characteristics between two different cluster subtypes in the training cohort, and the results revealed statistically significant differences in tumor purity, metastatic count, FGA and MSIsensor score, as determined by the Wilcoxon rank-sum test (*P*-value < 0.05; Figure 3B).

To comprehensively evaluate the characteristics of the tumor microenvironment, the activity levels of 114 metabolic pathways were compared between two different cluster subtypes in the training cohort. Significant differences were observed in the activity levels of 110 metabolic pathways (*P*-value < 0.05; Wilcoxon rank-sum test; Figure 3C). The t-distributed stochastic neighbor embedding (t-SNE) analysis was conducted to assess the activity level profiles of the two cluster subtypes. The results demonstrated apparent separation of pancreatic cancer cluster subtypes in the training cohort into two distinct components, with significant differences in activity levels between the cluster subtypes (Figure 3D). In order to investigate whether the cluster subtype was independent with other clinicopathologic characteristics, pancreatic cancer patients with available clinicopathologic characteristics included in a multivariate Cox regression analysis. As depicted in Figure 3E, the cluster subtype, sex, sample type and MSIsensor type were determined to be independent prognostic factors for overall survival.

A total of 433 pancreatic cancer patients from the testing cohort, with appropriately matched overall survival data and clinicopathologic data, were utilized to validate the cluster subtype model. The cluster subtype of each patient was determined by employing the set of 76 metabolic pathways identified in the training cohort and applying the SDCN algorithm. We explored cluster *k* values ranging from 2 to 10 and identified the optimal cluster by selecting *k* = 2, which exhibited the highest average silhouette coefficient (Figure S1C and D). The Kaplan–Meier survival curves demonstrated a significantly poorer overall survival in pancreatic cancer patients belonging to cluster 1 subtype when compared to those in cluster 2 subtype within the testing cohort (*P*-value = 4.99E-4; log-rank test; Figure S2A). Furthermore, the activity levels of the 114 metabolic pathways obtained in the testing cohort exhibited a similar pattern to that of the training cohort (Figure S2B). Multivariate Cox regression analysis conducted in the testing cohort demonstrated that the cluster subtypes derived from metabolic pathways, along with sample type and TMB, maintained their status as independent prognostic factors (Figure S2C).

### Enrichment analysis of two cluster subtypes

**Figure 4:**
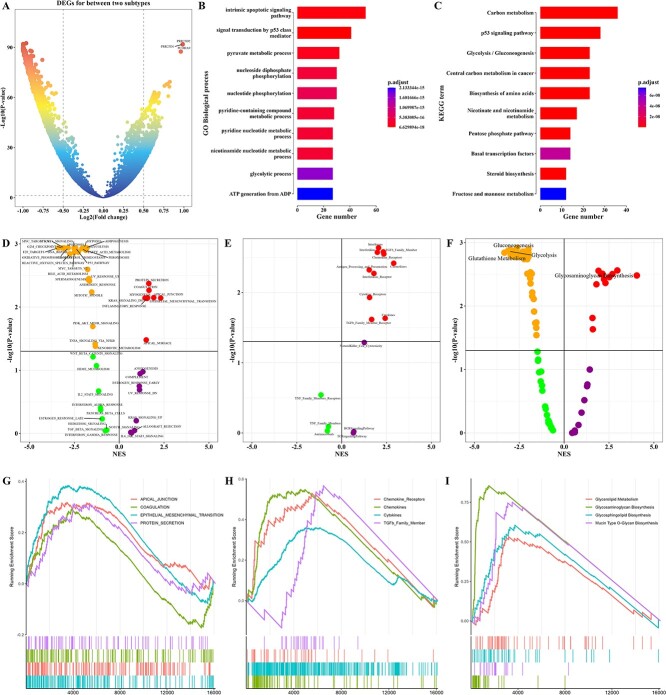
Biological characteristics of two cluster subtypes in the pancreatic cancer training cohort. (**A**) Volcano plot illustrated the differential genes between two cluster subtypes. The barplot illustrated the results of the enrichment analysis of differentially genes in (**B**) GO biological processes and (**C**) KEGG pathways. Volcano plots illustrated the GSEA results of (**D**) 50 hallmarks, (**E**) 17 ImmPort immune cell types and (**F**) 114 metabolic pathways between two cluster subtypes. GSEA plots were generated to visualize the enrichment results of the most significant 4 (**G**) hallmarks, (**H**) ImmPort immune cell types and (**I**) metabolic pathways between two cluster subtypes.

To investigate the potential mechanism between two cluster subtypes in the pancreatic cancer, the network propagation-based gene profile of 16 134 genes derived from the somatic mutation profile between two cluster subtypes in the training cohort were compared. A total of 754 differential genes between two cluster subtype samples (|Log_2_FC| ≥ 0.8 and *P*-value < 0.05) were obtained (Figure 4A). Functional enrichment analysis of GO and KEGG showed that these differential genes tended to be significantly enriched in biological processes and pathways associated with metabolism (Figure 4B and C).

To investigate the underlying mechanisms contributing to the disparate prognostic outcomes observed between the two cluster subtypes, a GSEA based on the hallmark gene set was conducted. As illustrated in Figure 4D and G, the patients with cluster 2 subtype displayed significant enrichment in hallmarks such as EPITHELIAL MESENCHYMAL TRANSITION, PROTEIN SECRETION, APICAL JUNCTION and COAGULATION, among others. On the other hand, the patients with cluster 1 subtype displayed significant enrichment in MYC TARGEMTSTOVR1C1 SIGNALING, E2F TARGETS, G2M CHECKPOINT, DNA REPAIR and more. To further compare the differences in the tumor immune microenvironment between the two cluster subtypes in the training cohort, a GSEA was performed using the ImmPort immune gene set. As a result, we found that patients with cluster 2 subtype were significantly enriched on 11 immune cell signatures, including chemokine receptors, chemokines, cytokines, TGFb family Member and so on (Figure 4E and H). In addition, Figure 4F and I illustrates the GSEA results of 114 metabolic pathways between the two cluster subtypes in the training cohort. Among these metabolic pathways, 87 were found to be significantly enriched between the two cluster subtypes.

### The landscape of the tumor immune microenvironment in different cluster subtypes

**Figure 5:**
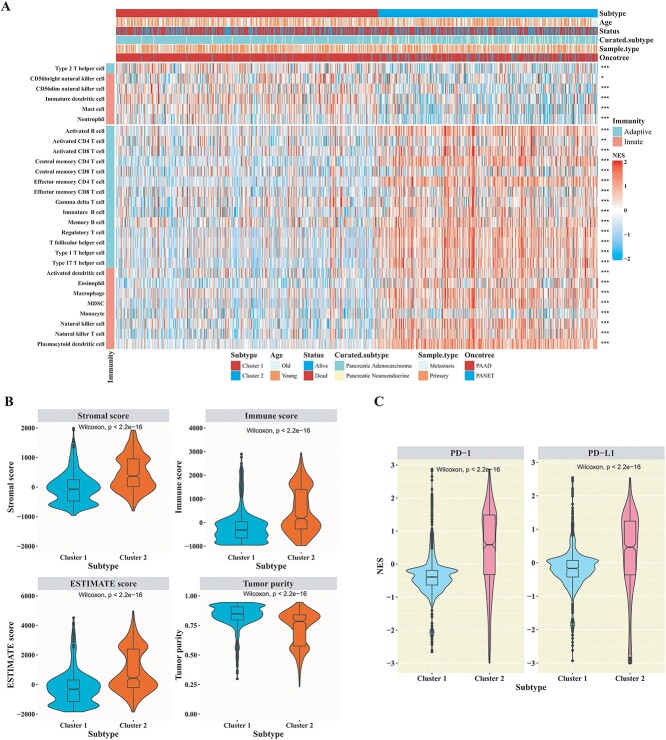
Immune landscape and immunotherapeutic potential of two cluster subtypes in the pancreatic cancer training cohort. (**A**) The NES distribution of 28 immune cell signatures between two cluster subtypes. (* indicated *P*-value < 0.05, ** indicated *P*-value < 0.01, *** indicated *P*-value < 0.001; Wilcoxon rank-sum test). (**B**) The violin plots distribution of stromal score, immune score, ESTIMATE score and tumor purity between two cluster subtypes. (Wilcoxon rank-sum test). (**C**) The violin plots distribution of PD-1 and PD-L1 between two cluster subtypes (Wilcoxon rank-sum test).

To comprehensively evaluate the landscape of tumor immune microenvironment in two different cluster subtypes in the training cohort, the infiltration levels of 28 immune cells were calculated from the smoothed mutation profile by using the ssGSEA. According to the Wilcoxon rank-sum test, significantly different immune infiltration levels were observed in the tumor immune microenvironment of two cluster subtypes, indicating that pancreatic cancer patients in different cluster subtypes exhibited distinct landscapes of immune cell infiltration (Figure 5A). As illustrated in Figure 5A, the infiltration levels of activated CD4 T cells, activated CD8 T cells, central memory CD4 T cells, central memory CD8 T cells, effector memory CD4 T cells and effector memory CD8 T cells were significantly higher in patients belonging to cluster 2 when compared to those in the cluster 1 subtype (*P*-value < 0.05; Wilcoxon rank-sum test; Figure 5A). In addition, patients belonging to the cluster 1 subtype exhibited higher infiltration levels of type 2 T helper cells, CD56bright natural killer cells, CD56dim natural killer cells, immature dendritic cells, mast cells and neutrophils compared to those in the cluster 2 subtype (*P*-value < 0.05; Wilcoxon rank-sum test; Figure 5A). Moreover, the estimate algorithm was employed to estimate the stromal score, immune score, ESTIMATE score and tumor purity. Significantly distinct values of these indices were observed between the two subtypes in the training cohort (*P*-value < 0.05; Wilcoxon rank-sum test; Figure 5B).

Immune checkpoints have been demonstrated to play crucial anti-tumor roles in cancer immunotherapy. The mRNA expression levels of immune checkpoints serve as valuable biomarkers in cancer immunotherapy. To compare the characteristics of immune checkpoints between the two cluster subtypes, we examined two well-known immune checkpoints: PD-1 and PD-L1. The NES levels of PD-1 and PD-L1 were illustrated using a network propagation–based gene profile with a violin plot (Figure 5C). As depicted in Figure 5C, the NES levels of PD-1 and PD-L1 showed a tendency to be higher in tumors of the cluster 2 subtype in the training cohort. Significantly, according to the Wilcoxon rank-sum test, the differences in NES levels of PD-1 and PD-L1 between the two cluster subtypes in the training cohort were statistically significant (*P*-value < 0.05; Figure 5C).

Furthermore, in this study, we employed CIBERSORT [57] to analyze the network propagation–based gene profile of pancreatic cancer patients in the training cohort, obtaining the infiltration proportions of 22 immune cell types in the pancreatic cancer immune microenvironment (Figure S3A). Additionally, we performed a comparative analysis of these proportions between the two cluster subtypes, revealing significant differences in 14 immune cell types (*P*-value < 0.05; Wilcoxon rank-sum test; Figure S3B).

### Immunotherapy response in different cluster subtypes

To further evaluate the universal applicability of the cluster subtype in predicting responsiveness to immunotherapy, we assessed the performance of the TIDE algorithm using pancreatic cancer samples from the training cohort (Figure S4A). Interestingly, the cluster 2 subtype demonstrated significantly lower scores in TIDE, MSI Score, exclusion, MDSC and TAM.M2 compared to the cluster 1 subtype (*P*-value < 0.05; Wilcoxon rank-sum test; Figure S4A). Moreover, the cluster 2 subtype exhibited significantly higher scores of dysfunction when compared to the cluster 1 subtype (*P*-value < 0.05; Wilcoxon rank-sum test; Figure S4A).

We observed a significantly higher proportion of patients in the cluster 2 subtype who responded to ICB treatment compared to the cluster 1 subtype patients (*P*-value < 2.2e-16; chi-square test; Figure S4B). These findings strongly suggest that cluster subtypes can serve as a valuable tool for predicting the therapeutic efficacy of immunotherapy in patients with pancreatic cancer.

### XGBoost classifier for prediction of pancreatic cancer patients with different cluster subtypes

In this study, our objective was to construct a classifier capable of predicting the cluster subtypes of pancreatic cancer patients based on the activity levels of 76 prognostic-related metabolic pathways. To accomplish this, we employed the XGBoost (eXtreme Gradient Boosting) classifier [58, 59], implemented in the Python package xgboost (version 1.7.6), to develop a classifier that could distinguish between cluster 1 subtype and cluster 2 subtype. The optimal parameters for XGBoost were determined using the GridSearchCV function from the Python package scikit-learn (version 1.2.2) [60] during 10-fold cross-validation. Based on the activity levels of 76 metabolic pathways, we achieved an overall accuracy of 97.87% through 10-fold cross-validation in the training cohort. This predictive result indicated that the XGBoost algorithm was suitable for identifying the cluster subtypes for patients with pancreatic cancer.

**Figure 6:**
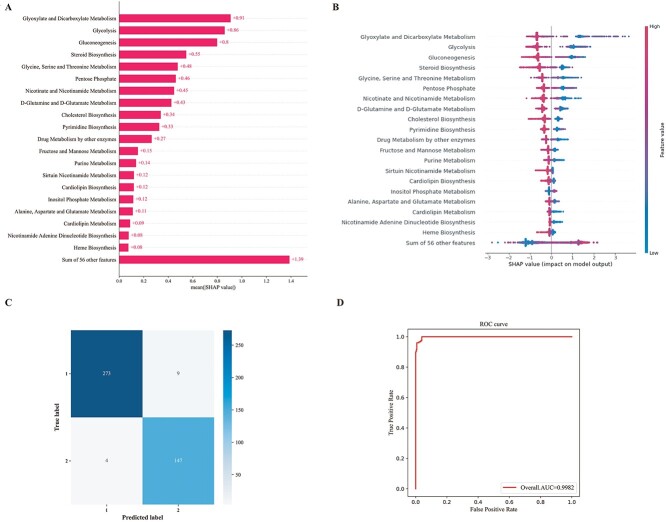
Prediction of pancreatic cancer patients with different cluster subtypes by XGBoost algorithm. (**A**) The mean absolute SHAP values of the top 20 metabolic pathways. (**B**) Distribution of the SHAP values for the top 20 metabolic pathways based on the highest mean absolute SHAP value. The *x*-axis illustrated the SHAP value. The colors of the dots corresponded to the magnitude of their respective observed values for metabolic pathways, ranging from small to large or negative to positive. (**C**) Confusion matrix for prediction of pancreatic cancer patients in the testing cohort. (**D**) The ROC curve of XGBoost model for prediction of pancreatic cancer patients in the testing cohort.

To gain deeper insights into the relative contributions of the metabolic pathways in the XGBoost algorithm, SHapley Additive exPlanations (SHAP) analysis was employed. The top 20 metabolic pathways with descending importance on the model output in the training cohort according to the mean absolute SHAP values and SHAP values are illustrated in Figure 6A and B. In the training cohort, we found that the model’s most predictive power was carried by glyoxylate and dicarboxylate metabolism; glycolysis; gluconeogenesis; steroid biosynthesis; glycine, serine and threonine metabolism; pentose phosphate pathway; and nicotinate and nicotinamide metabolism, as well as D-glutamine and D-glutamate metabolism. All of these metabolic pathways illustrated a positive impact on the predictive model. In addition, these top metabolic pathways identified through SHAP analysis in the XGBoost algorithm may serve as risk signatures distinguishing between the two metabolic subtypes in pancreatic cancer.

Since the XGBoost model had the powerful ability to predict the cluster subtypes of pancreatic cancer patients, the XGBoost model constructed from the training cohort was applied to predict the patient cluster labels in the testing cohort. The predicted results indicate that out of the 282 pancreatic cancer patients in cluster 1 subtype, 273 were correctly classified, while out of the 151 pancreatic cancer patients in cluster 2 subtype, 147 were correctly classified (Figure 6C). The predicted AUC curve area was 0.9982 (Figure 6D). These results demonstrate that our XGBoost classifier can accurately predict the category of pancreatic cancer patients with unknown subtypes.

### Validation of our method on different cancer cohorts

Given the clinically meaningful and prognostic subtypes in pancreatic cancer, we aimed to apply this algorithm to 11 independent MSKCC cancer cohorts to classify patients into distinct molecular subtypes to assess the robustness and practical application of our algorithm. We utilized the same algorithm for each MSKCC cancer cohort to calculate the activity levels of 114 metabolic pathways for every cancer patient. Univariate Cox regression analysis was conducted to identify the metabolic pathways with prognostic significance (*P*-value < 0.05; log-rank test). Subsequently, the SDCN algorithm was applied to identify robust clusters for each MSKCC cancer cohort, based on the prognostic-related metabolic pathways. Univariate Cox regression analysis was performed on the cluster subtypes stratified by the SDCN for each of the MSKCC cancer cohorts. In all 11 independent MSKCC cancer cohorts, there were significant associations between clustering subtypes and prognosis (*P*-value < 0.05; log-rank test; Figure S5). These findings demonstrated the robustness of our algorithm in diverse MSKCC cancer cohorts.

In addition, we wanted to investigate the effect of randomized somatic mutation profiles on stratification of MSKCC pancreatic cancer patients by using the same algorithm. First, we should construct a randomized somatic mutation profile. To accomplish this, we performed sampling with replacement on patient mutation profiles from the pancreatic cancer cohort. Subsequently, we permuted the mutation profile for each patient across the entire gene panel, while preserving the per-patient mutation frequency, resulting in a randomized mutation matrix devoid of any biological signal. We used the same algorithm to calculate the activity levels of 114 metabolic pathways for the randomized mutation profile. Through univariate Cox regression analysis, we identified 12 metabolic pathways significantly associated with prognosis (*P*-value < 0.05; log-rank test; Figure S6A). Based on these 12 prognostic-related metabolic pathways, we performed deep clustering to classify all pancreatic cancer patients into two molecular subtypes (Figure S6B and C). However, according to the log-rank test, no significant difference was found between the two pancreatic cancer subtypes generated by the randomized mutation profile (Figure S6D). These findings indicated that the actual somatic mutation data of pancreatic cancer from MSKCC have significant biological relevance compared with the randomized mutation profile.

To evaluate the applicability of our algorithm in other cancer datasets, we further validated the prognostic potential of this model in the TCGA pancreatic adenocarcinoma (PAAD) cohort. Employing the same methodology, we identified 143 TCGA PAAD patients classified into two distinct molecular subtypes. Kaplan–Meier survival curves demonstrated a significant association between cluster 1 subtype and poorer prognosis compared to cluster 2 subtype within the TCGA PAAD cohort (*P*-value = 1.99E-2; log-rank test; Figure S7).

### Integrative construction and evaluation of machine learning–based MRS

**Figure 7:**
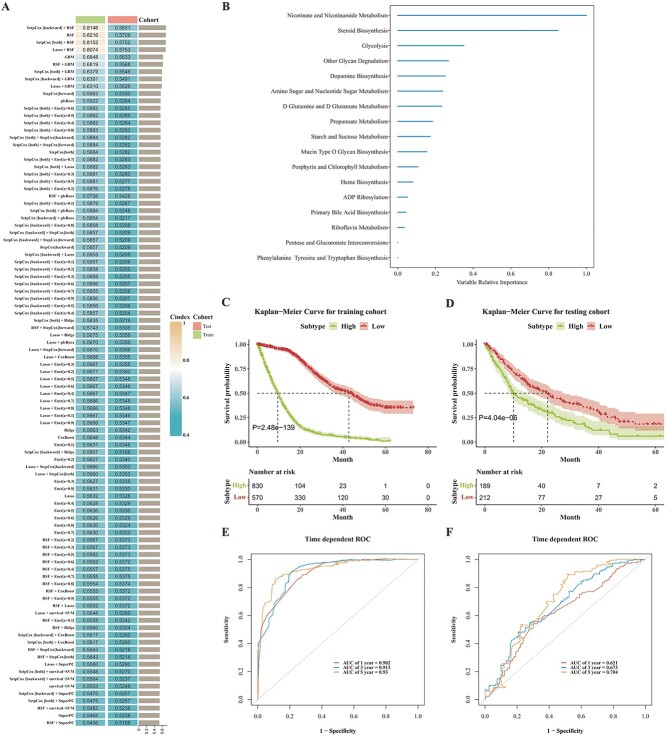
Construction and evaluation of machine learning-based MRS. (**A**) The C-indices of 96 machine learning algorithms in the training and testing cohorts. (**B**) The variable relative importance of 17 metabolic pathways in the RSF. (**C**) Kaplan–Meier curves for overall survival between the high and low machine learning–based MRS subtypes in the training cohort and testing cohort. The time dependent ROC for predicting 1, 3 and 5 year overall survival in the (**E**) training cohort and (**F**) testing cohort.

The activity levels of 76 prognostic-related metabolic pathways underwent our machine learning–based integrative procedure to construct an MRS. A total of 96 machine learning models were employed on the pancreatic cancer training cohort using 10-fold cross-validation to develop prediction models. The average C-index of each model was calculated across both the training and testing cohorts (Figure 7A). As depicted in Figure 7A, the combination of SetpCox [backward] and RSF, which yielded the highest average C-index (0.699), was chosen as the final model. By employing RSF, a set of 17 metabolic pathways was identified and utilized to construct the machine learning–based MRS for each patient within the training and testing cohorts (Figure 7B).

All pancreatic cancer patients in both the training and testing cohorts were categorized into high- and low-risk subtypes based on the optimal cut-off value determined using the R package survminer (version 0.4.9). Kaplan–Meier survival analysis revealed that the low-risk subtype exhibited a significantly longer overall survival time compared to the high-risk subtype in both the training cohort (*P*-value = 2.48E-139; log-rank test; Figure 7C) and the testing cohort (*P*-value = 4.04E-6; log-rank test; Figure 7D).

To evaluate the discriminative ability of the MRS, receiver-operating characteristic (ROC) curves were generated for both the training and testing cohorts. The areas under the ROC curve (AUCs) were calculated for 1, 3 and 5 year overall survival. In the training cohort, the AUCs for 1, 3 and 5 year overall survival were 0.902, 0.913 and 0.930, respectively (Figure 7E). In the testing cohort, the corresponding AUCs were 0.621, 0.673 and 0.704, respectively (Figure 7F). These AUC values, obtained from both the training and testing cohorts, indicate that the MRS exhibits favorable predictive performance in estimating the overall survival of pancreatic cancer patients.

### Establishment the nomogram model for the machine learning–based MRS

Although our machine learning–based MRS model can predict the survival probability of pancreatic cancer patients, this risk prediction approach still faces challenges in achieving personalized accuracy. Moreover, there is a need for a model that can effectively integrate both the MRS and clinicopathological factors to maximize the utilization of prognostic information from patients.

To provide a quantitative method for predicting the probability of overall survival, we constructed a nomogram model that integrated both the MRS and clinicopathological factors using pancreatic cancer patients from the training and testing cohorts (Figures S8A and S9A). As depicted in Figures S8A and S9A, a higher MRS in the nomogram was associated with a worse 1, 3 and 5 year overall survival probability in both the training and testing cohorts. The MRS exhibited a greater contribution to the risk points compared to the other clinicopathological factors in both cohorts (Figures S8A and S9A).

The calibration curves of overall survival demonstrated agreement between the predicted overall survival probability by the nomogram and the observed overall survival probability at 1, 3 and 5 year time points (Figures S8B and S9B), indicating the reliability of this nomogram in survival prediction. The decision curve analysis showed that the nomogram provided greater net benefits across a range of threshold probabilities compared to either the treat-all-patients or treat-none schemes in both the training and testing cohorts (Figures S8C and S9C).

## DISCUSSION

Pancreatic cancer is an extremely aggressive malignant neoplasm that arises from malignant cells in the pancreatic tissue, ranking among the most lethal malignancies globally. The 1-year survival rate is merely 25%, while the 5-year survival rate drops even lower to 10%. In 2020, a total of 495 773 new pancreatic cancer cases were diagnosed globally, leading to approximately 466 003 deaths [2]. Projections indicate that by 2030, pancreatic cancer is projected to be the second leading cause of cancer-related mortality. Pancreatic cancer exhibits significant heterogeneity, making accurate stratification of the disease an essential step in guiding precise treatment decisions. Stratifying pancreatic cancer with somatic mutations may aid in identifying patient subtypes that could benefit from targeted treatment. Unlike the mRNA expression profile and other omics profiles, somatic mutations are exclusively differential measurements between tumor and normal tissue. Consequently, a quantitative value cannot be assigned to each patient. Thus, it is very challenging to stratify pancreatic cancer patients solely based on somatic mutation profile. In this study, we developed an algorithm that integrated the somatic mutation profile with network propagation for classifying pancreatic cancer subtypes. Our study demonstrated that pancreatic cancer subtypes were correlated with prognosis and clinical phenotypes and may offer potential targeted treatment options for specific subtypes of pancreatic cancer.

Next-generation sequencing assays have led to the development of MSK-IMPACT, which was originally designed by Cheng *et al.* for the efficient and precise identification of somatic mutations, copy number alterations and structural variants in patient tumors [30]. In their study, Cheng *et al.* employed a validation set comprising 284 distinct tumor samples with known SNVs and indels, which had been previously confirmed using independent methods. Additionally, they utilized an independent set of selected samples to demonstrate that MSK-IMPACT can effectively detect known somatic mutations, copy number alterations and structural variants in tumor samples with a high degree of comprehensiveness, accuracy, sensitivity and reproducibility. Drawing from these findings, they concluded that MSK-IMPACT can be comprehensively utilized to inform treatment decisions for patients with tumors.

In a separate research project, Cheng *et al.* conducted additional evaluations of MSK-IMPACT’s performance in detecting germline variants within a subset of 76 genes associated with cancer predisposition syndromes [31]. Their findings revealed that MSK-IMPACT successfully identified all germline variants within a collection of 233 distinct patient DNA samples, which had previously been validated through single gene testing. Additionally, they discovered further pathogenic mutations beyond those previously identified using conventional methods. This study underscores the significance of the MSK-IMPACT approach in comprehensively detecting germline variants in associated with cancer predisposition syndromes and simultaneous detection of somatic and germline alterations.

In the work of Fiala *et al.*, they utilized the MSK-IMPACT platform to analyze 751 pediatric solid tumor patients, identifying mutations, copy number alterations and gene fusions [32]. Their findings, based on the MSK-IMPACT platform, revealed the prevalence and spectrum of pathogenic and likely pathogenic (P/LP) germline variants in genes associated with cancer predisposition. They also uncovered associations between germline genetic status and somatic molecular profiles, highlighting the clinical relevance of this information.

In the work of Nguyen *et al.*, they collected a large clinico-genomic cohort from the MSKCC that covering more than 25 000 cancer patients with metastasis across 50 tumor types [33]. By analyzing genomic and clinical data from this cohort, they identified associations between genomic alterations and patterns of metastatic dissemination across the 50 tumor types. Correlations between chromosomal instability and metastatic burden were observed in specific tumor types. Additionally, somatic alterations were identified to be correlated with both metastatic burden and specific target organs.

Due to the high dimensionality and sparsity of somatic mutations identified by the MSK-IMPACT platform, it is not easy to use these somatic mutations. In this study, we proposed the network propagation algorithm to transform the binary vectors of somatic mutations into continuous vectors of mutational statuses for MSKCC pancreatic cancer patients. Because the smoothed mutation profile was similar to the mRNA profile, a variety of bioinformatics analysis methods, including enrichment analysis, GSVA and GSEA, were performed on this smoothed mutation profile. Researchers can also utilize the smoothed mutation profile constructed by our network propagation algorithm to classify MSKCC cancer patients into clinically and biologically meaningful subtypes, which may not be easily achieved with sparse somatic mutations alone. These distinct subtypes classified by our algorithm may provide more precise outcome predictions, additional insights into the selection of optimal therapies and a better understanding of the heterogeneity among MSKCC cancer patients.

To assess the clinical relevance of our established metabolic subtypes using our network propagation model, we investigated the association between these subtypes and prognosis in pancreatic cancer. The results revealed a significant difference in prognosis between the two metabolic subtypes. Additionally, we compared six clinicopathological characteristics, namely, tumor purity, metastatic count, metastatic site count, TMB, FGA and MSIsensor score, between the two metabolic subtypes. The results assessed using the Wilcoxon rank-sum test demonstrated statistically significant differences in tumor purity, metastatic count, FGA and MSIsensor score. These aforementioned results clearly establish the clinical relevance of the metabolic subtypes established in our study. The biological relevance of the metabolic subtypes was assessed through functional enrichment analysis of GO and KEGG, as well as GSEA of the hallmark gene set, ImmPort immune gene set and metabolic pathways. All the analysis results unambiguously demonstrated the biological relevance of the metabolic subtypes established in our study.

Metabolism plays a central role in all biological processes, and disruptions in metabolic regulation are a defining characteristic of numerous conditions, such as cancer, diabetes and cardiovascular diseases. In recent years, a lot of experimental and computational methods, including mass spectrometry, pathway-based analysis and flux balance analysis (FBA)–based methods, have been developed to detect metabolic pathways. Compared with these methods, our method does not rely on experimental methods and gene expression profiles; it can effectively measure metabolic pathways solely using somatic mutation profiles.

Through our study, we have discovered that by applying a network propagation algorithm to smooth somatic mutation profiles and subsequently utilizing the GSVA algorithm, we can obtain activity levels of metabolic pathways. Furthermore, we have found that the activity levels of metabolic pathways obtained through this study are significantly associated with the prognosis of pancreatic cancer patients. Using the SDCN clustering algorithm, we can identify metabolic subtypes of pancreatic cancer that are biologically meaningful and clinically relevant.

Compared to other experimental and computational methods for measuring metabolic pathways, the method proposed in this study represents the first instance where it is shown that biologically meaningful and clinically relevant metabolic pathways can be obtained solely relying on somatic mutation profiles of pancreatic cancer. The findings of this study hold significant importance to the classification of metabolic subtypes using cancer somatic mutation profiles.

Cancer is fundamentally caused by the accumulation of somatic mutations in cells. In normal human somatic cells, there are generally a significant number of somatic mutations, and as age advances, these mutations continue to accumulate within cells. When the mutations accumulate to a certain extent, they can trigger changes in the overall tissue function and morphology, leading to the development of cancer. Therefore, the somatic mutation profile can capture the causal genetic events underlying tumor progression. Utilizing somatic mutation profiles may improve the accuracy, reproducibility and robustness to noise in identifying metabolic subtypes of pancreatic cancer patients. The significance of this study lies not only in filling the gap in research related to metabolic profiling using somatic mutation data of pancreatic cancer patients but also in further enriching and refining our understanding of metabolism. This contributes to the goal of reducing the mortality rate and improving the quality of life for pancreatic cancer patients.

To establish a classification scheme for pancreatic cancer based on tumor driver genes, we retrieved 64 pancreatic cancer driver genes from the IntOGen database (Release 2023-05-31) [61]. This database is dedicated to collecting and analyzing somatic mutations in thousands of tumor genomes for the identification of cancer driver genes. Because mutation data consisting solely of 0 and 1 are not suitable for clustering, we utilize the smoothed somatic mutation profiles generated in our study for the classification of 1933 MSKCC pancreatic cancer patients by driver genes. According to the smoothed somatic mutation profiles of 64 driver genes, 1933 MSKCC pancreatic cancer patients were classified into two different clustering subtypes (Figure S10A and B). The Kaplan–Meier curve demonstrated that the patients in cluster 1 subtype possessed significantly longer overall survival time than those in cluster 2 subtype in the MSKCC pancreatic cancer cohort (*P*-value = 1.14e-05; Figure S10C). We then investigated the relationship between metabolic subtypes and driver gene classification. From this Sankey diagram, we noticed that most of the patients in cluster 1 metabolic subtype displayed the cluster 2 driver genes subtypes (Figure S10D). The metabolic subtypes established by our study may be different from driver gene classification in the MSKCC pancreatic cancer patients.

While it is possible to classify pancreatic cancer directly using driver genes, this approach does not necessarily associate pancreatic cancer subtypes with specific immune or metabolic pathway subtypes. Our elaborative method, established here, addresses the challenge of discrete mutation data in cancer by applying a network propagation algorithm to smooth the data. Additionally, by utilizing metabolic pathways for classification in this study, we can obtain pancreatic cancer metabolic subtypes that are more biologically relevant, bridging the gap between pancreatic cancer classification and metabolic pathways. This approach can potentially contribute to the study of pancreatic cancer metabolism.

Generally, the clinical staging of pancreatic cancer (standard classification) is typically based on criteria such as tumor size, depth of invasion, lymph node involvement and the presence of distant metastasis. This staging is often described using the International Cancer Staging System (TNM system). However, in our study, specific TNM stage information was not available for our MSKCC pancreatic cancer cohort. Therefore, as an alternative, we compared the metabolic subtypes we had constructed with other classifications of pancreatic cancer, such as tumor driver classification, OncoTree classification and primary or metastasis classification. In cancer research, Sankey diagrams can be used to compare the similarities and differences between different cancer classifications. Therefore, in this study, we used Sankey diagrams to compare the metabolic subtypes of pancreatic cancer obtained in our research with the OncoTree classification and primary or metastasis classification (Figure S11). Through Sankey diagrams, it is possible to clearly visualize the connections between different cancer classifications and understand their commonalities and differences. This assists researchers and clinicians in better understanding the biological and clinical distinctions between different classifications, thereby providing more accurate decision-making for personalized treatment and prognosis assessment.

The main contribution of this work is that for the first time, our work presented that using solely on somatic mutation data to classify MSKCC cancer patients into clinically and biologically meaningful metabolic subtypes. Seventy-six metabolic pathways were found to have significant associations with the prognosis of MSKCC cancer patients. We employed these 76 metabolic pathways to construct an XGBoost algorithm, yielding favorable outcomes. The top 20 metabolic pathways, such as glyoxylate and dicarboxylate metabolism, glycolysis, gluconeogenesis and steroid biosynthesis, identified through SHAP analysis in the XGBoost algorithm, may serve as risk signatures distinguishing between the two metabolic subtypes in pancreatic cancer (Figure 6A and B).

Although our somatic mutation-based stratification algorithm was promising, some limitations should be acknowledged in this study. Firstly, all the cancer samples used in this study were obtained from the MSKCC database. However, further validation of this algorithm should be conducted using other types of somatic mutation databases. Secondly, the disparity in the biological characteristics landscape between the two pancreatic cancer subtypes observed in this study should be corroborated with mRNA data or other omics data, rather than relying solely on data derived from somatic mutations. Thirdly, the predictive data for immunotherapy and chemotherapy also stem from somatic mutation data, and further validation necessitates clinical pancreatic cancer cohorts with immunotherapeutic information. Finally, the construction of the biological network in this investigation solely relied on the utilization of the STRING database, which primarily captures protein–protein interactions. While the STRING network demonstrated commendable performance in the network propagation model, exploring alternative types of protein–protein interaction networks is advisable. Such networks encompass biological signaling, metabolism, or transcription information and have the potential to enhance the network propagation model’s overall performance.

We developed a network propagation-based pipeline to integrate somatic mutation profiles, stratifying pancreatic cancer patients into two molecular subtypes with distinct biological characteristics and clinical outcomes. Our study may enhance the understanding of pancreatic cancer heterogeneity, facilitate clinical stratified management and enable precise treatment for pancreatic cancer patients. In the future, the efficacy of our study may be enhanced through the inclusion of more clinically significant somatic genes and the utilization of more suitable protein interaction networks.

**Key Points**

- A network propagation model was constructed to smooth mutation profiles of pancreatic cancer.
- A structural deep clustering network was utilized to establish biologically and clinically relevant metabolic subtypes of pancreatic cancer
- Metabolic subtypes reveal distinct immunogenomic landscapes, therapeutic implications and other pertinent factors.

## Supplementary Material

supplementary_material_bbad430

## Data Availability

The datasets supporting the conclusions of this article are available in the cBioPortal database (https://www.cbioportal.org/study?id=msk_met_2021). The raw experimental data supporting the conclusions of this article will be made available by the corresponding author. The source codes employed in this study have been made accessible and are available for reference at the following GitHub repository: https://github.com/LeiyangHarbin/BIB.

## Author Biographies

**Min Zou** is a PhD student at the College of Bioinformatics Science and Technology, Harbin Medical University. Her research interests include deep clustering and metabolism genomics.

**Honghao Li** is a master student at the College of Bioinformatics Science and Technology, Harbin Medical University. His research interests include somatic mutation and structural deep clustering network.

**Dongqing Su** is a PhD student at the College of Bioinformatics Science and Technology, Harbin Medical University. Her research interests include computational biology and comparative genomics.

**Yuqiang Xiong** is a master student at the College of Bioinformatics Science and Technology, Harbin Medical University. His research interests include disease biomarker detection and data mining.

**Haodong Wei** is a master student at the College of Bioinformatics Science and Technology, Harbin Medical University. His research interests include clustering algorithm and transcription regulation.

**Shiyuan Wang** is an associate professor at the College of Bioinformatics Science and Technology, Harbin Medical University. Her research interests include computational biology and integration analysis of the metabolic pathway.

**Hongmei Sun** is the associated research fellow at the College of Bioinformatics Science and Technology, Harbin Medical University. Her research interests include comparative genomics and computational biology.

**Tao Wang** is a master student at the College of Bioinformatics Science and Technology, Harbin Medical University. His research interests include tumor microenvironment and radiology.

**Qilemuge Xi** is a lab master at the College of Life Sciences, Inner Mongolia University. Her research interests include genomics and the functional diversity of proteins.

**Yongchun Zuo** is a professor at the State Key Laboratory of Reproductive Regulation and Breeding of Grassland Livestock, College of Life Sciences, Inner Mongolia University. His research interests include bioinformatics and multi-omics integration analysis in cell reprogramming.

**Lei Yang** is a professor at the College of Bioinformatics Science and Technology, Harbin Medical University. His research interests include machine learning for improving healthcare and bioinformatics.
